# Relationship Between Breast Cancer Risk and Polymorphisms in *CLOCK* Gene: A Systematic Review and Meta-Analysis

**DOI:** 10.1007/s10528-023-10372-2

**Published:** 2023-04-10

**Authors:** Yi Shi, Lixing Wu, Xuenian Ji, Yunru Li, Zhicheng Zhang

**Affiliations:** 1 Dongzhimen Hospital, Beijing University of Chinese Medicine, Beijing, China; 2 Ningjin Hospital of Chinese Medicine, Shandong, China; 3 Beijing University of Chinese Medicine, Beijing, China

**Keywords:** Breast cancer, CLOCK gene, Polymorphisms, Meta-analysis

## Abstract

Previous studies found that the circadian clock gene participated in the genesis and development of breast cancer. However, research findings on the relationship between polymorphisms in the *CLOCK* gene and breast cancer risk were inconsistent. This study performed a meta-analysis of the association between *CLOCK* gene polymorphisms and breast cancer risk. PubMed, Cochrane Library, and Embase databases were electronically searched to collect studies on the association between *CLOCK* gene polymorphisms and breast cancer risk from inception to February 14, 2022. The quality of the included literature was assessed using the Newcastle–Ottawa Scale. For statistical analysis, odds ratio (OR) and 95% confidence intervals (CIs) were calculated using STATA 14.0. In addition, publication bias was performed by the funnel diagram and the Harbord’s regression test. And sensitivity analysis was assessed by the trim and fill method. A total of 6 eligible studies, including 10,164 subjects (5488 breast cancer cases and 4676 controls), were screened in this meta-analysis. Though we did not find a significant association between the polymorphisms in the overall *CLOCK* gene with breast cancer risk [OR (95%CI) = 0.98 (0.96, 1.01), *P* = 0.148], we found that compared with T/T types of rs3749474 in *CLOCK*, T/C and C/C types of rs3749474 were associated with lower risk of breast cancer [OR (95%CI) = 0.93 (0.88, 0.98), *P* = 0.003]. The sensitivity analysis confirmed the robustness of the results. The funnel plot showed no significant publication bias. Polymorphisms in the *CLOCK* gene might be associated with breast cancer risk. More studies are needed to confirm the conclusion.

## Introduction

Breast cancer is one of the most common malignancies in women worldwide, and its incidence is increasing yearly (Nounu et al. 2022). It was reported that there were 281,550 diagnosed cases of breast cancer around the world in 2021, and breast cancer was estimated to cause nearly 40,000 deaths, which accounted for 7% of all cancer mortality each year (Ropri et al. 2021). The traditional risk factors of breast cancer include reproductive cycle, lifestyle, obesity, genetic susceptibility, DNA methylation, miRNA, and family history (Cheng et al. 2022; Jiralerspong et al. 2009; Joo et al. 2018; Li et al. 2010; Lin et al. 2022; Wang et al. 2022; Wen et al. 2022; Zhou et al. 2014). These days, large numbers of epidemiological studies have shown that circadian rhythm disruption, which is considered carcinogenic to humans (group 2A), contributes to the increased incidence of breast cancer (Dieterich et al. 2014; Samuelsson et al. 2018). Using data from the Nurses’ Health Study with 0.19 million participants, Wegrzyn et al. found that 20 years or more of night-shift work was associated with a significantly higher risk of breast cancer compared with non-night shift workers [HR(95%CI) = 2.15 (1.23, 2.73)], highlighting the importance of circadian rhythm in the pathogenesis of breast cancer (Wegrzyn et al. 2017).

The circadian rhythm is generated and controlled by a series of circadian genes involved in maintaining the internal coordination of multiple oscillators within and between various organ systems to provide the most efficient response to the day/night cycle (Bass and Takahashi 2010). These genes include *basic helix-loop-helix ARNT like 1* (*BMAL1*), *Circadian Locomotor Output Cycles Kaput* (*CLOCK*), *cryptochrome circadian regulator 1/2*, *period circadian regulator 1/2/3*. (Masri and Sassone-Corsi 2018). Among them, the *CLOCK* gene was discovered as the first mammalian circadian gene, located in human chromosome 4q12; it codifies the CLOCK protein, a positive regulatory arm of the circadian system (Pagliai et al. 2019). CLOCK plays a role in regulating cell physiological processes, such as the cell cycle, DNA damage response, cell proliferation, and apoptosis. Most epidemiology studies have indicated that *CLOCK* gene variations are associated with the risk of obesity, cardiovascular diseases, type 2 diabetes, and different types of cancer (Cuninkova and Brown 2008; Valenzuela et al. 2016). However, studies on the effect of *CLOCK* polymorphisms on breast cancer yield inconsistent results. Zienolddiny et al. found that TT carriers in *CLOCK* rs3749474 had a reduced risk of breast cancer [OR (95%CI) = 0.64 (0.45, 0.92)] among 563 breast cancer cases and 619 controls (Zienolddiny et al. 2013), while Hoffman et al. did not find the significant association between *CLOCK* rs3749474 and breast cancer risk (Hoffman et al. 2010). Therefore, the need for further systematical research on the effects of *CLOCK* polymorphisms on breast cancer remains.

In this study, we conducted a meta-analysis to systematically evaluate the associations between *CLOCK* gene polymorphisms and breast cancer risk.

## Methods

### Literature Search Strategy

We prepared this report in accordance with the Preferred Reporting Items for Systematic Reviews and Meta-analyses (PRISMA) principle (Liberati et al. 2009). Two researchers, Lixing Wu, and Xuenian Ji, independently searched PubMed, Cochrane Library, and Embase databases, and the search period was set from inception through February 14, 2022. The literature search was conducted according to the search characteristics of each database, and the following keywords were used: ("sleep–wake circadian rhythms" OR "circadian sleep disorders" OR "sleep–wake pattern" OR" sleep disorders" OR "circadian rhythm2 disorder" OR "circadian rhythm") AND ("clock gene" OR "CLOCK") AND ("breast cancer" OR "breast tumor" OR "breast carcinoma"). We first screened the titles  and abstracts, then read the full text of all potentially eligible studies. The references of the included literature were also manually searched and reviewed for additional literature.

### Inclusion and Exclusion Criteria

Inclusion criteria were as follows: (1) patients definitively diagnosed with breast cancer; (2) definitive detection of CLOCK genotype and allele frequency that were provided in the study; (3) relationships between *CLOCK* gene polymorphism and the risk of breast cancer that were evaluated; (4) odds ratio (OR), hazard ratio and 95% confidence intervals that could be obtained directly or indirectly calculated based on the data provided in the graphics or tables; (5) study in English.

Exclusion criteria were as follows: (1) duplicate publications or incomplete data; (2) reviews, meta-analysis, only abstracts, conference proceedings, expert reviews, and case reports; (3) literature with unclear diagnostic criteria of breast cancer and genotyping methods; (4) control group with unclear population or history of cervical cancer-related diseases; (5) cell or animal studies.

### Data Extraction

Two investigators independently screened the literature and extracted the required information from the literature. The required information included: first author, year of publication, geographic region, *CLOCK* polymorphisms, sample size, clinical stage, detection method, age, and the number of subjects.

### Quality Evaluation

Two researchers independently evaluated the quality of the included literature according to the Newcastle–Ottawa Scale (NOS) criteria and the total score ranging from 0 to 9 (Stang 2010). Studies with a total NOS score ≥ 6 are considered high-quality studies (Xiong et al. 2018).

### Statistical Analysis

In this meta-analysis, the pooled odds ratio (OR) with its corresponding 95% confidence intervals (CIs) were used to assess the effect size for studies that reported the association between *CLOCK* gene polymorphism and the risk of breast cancer. The dominant model, the recessive model, and the over-dominant model were used in the meta-analysis. We used the Q test and I^2^ statistic to evaluate the heterogeneity across studies. If *P* ≥ 0.1 and I^2^ < 50%, the results indicated that the homogeneity among studies was good, and the fixed-effect model was chosen for the meta-analysis; if *P* < 0.1 and I^2^ ≥ 50%, the results indicated that there was heterogeneity among studies, and the random-effect model was chosen. Sensitivity analysis was used to evaluate the effect of individual studies on the final results and to determine the reliability of the results. Funnel plots and Harbord’s test was used to assess whether there was significant publication bias. Meta-analyses were performed using STATA 14.0 (Stata Corporation, College Station, Texas, USA). Except for the heterogeneity test, two-sided *P* values < 0.05 were considered statistically significant.

## Results

### Literature Search

**Fig. 1:**
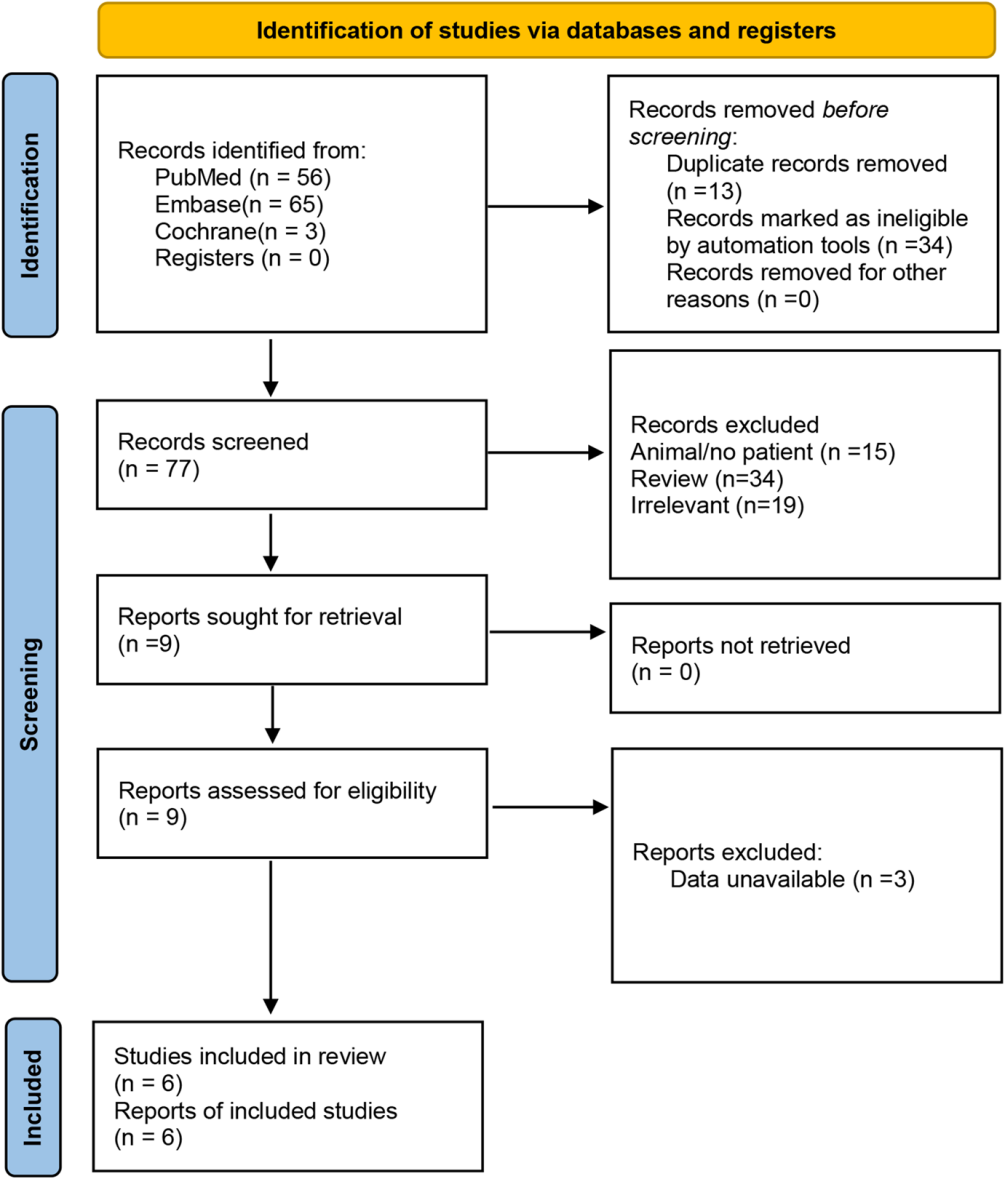
Flowchart illustrating the article selection process according to the PRISMA guidelines

According to the search strategy, a total of 122 studies were obtained from various databases. Among them, 13 studies were excluded due to duplication; 34 studies were removed due to being reviews, only abstracts, conference literature, or systematic evaluation; 15 animal experiments were excluded; 19 studies with non-specified genes were excluded. Therefore, seven studies were obtained provisionally. In addition, two studies were obtained from reference list searching. After excluding three studies with incomplete data, six studies comprising 10,164 subjects, including 5488 breast cancer cases and 4676 controls, remained in the meta-analysis. The literature search process is detailed in Fig. 1. All 6 studies were case–control studies, and the genotypes of *CLOCK* were determined by Polymerase Chain Reaction (PCR). Characteristics of the included 6 studies are shown in Table 1. In addition, the NOS quality scores of these studies ranged from 6 to 8 points (Table 2).

**Table1:** Characteristics of the included studies in the meta-analysis

Study	Country	Design	Gene type	Sample size	Detection	Diagnostic	age	Outcome
Aaron E. Hoffman, 2010	USA	case–control	rs7698022;rs6850524;rs11133391;rs11133389;rs13102385;rs11932595;rs1801260;rs3749474;rs1048004;rs3805151;	441 + 479	PCR	International Classification of Diseases for Oncology, 174.0–174.9	30–80	Breast cancer
Hongji Dai,2010	China	population-based case–control	rs3805151	1538 + 1605	PCR	Not mentioned	≤ 50y 47.2%; > 50y 52.8%	primary breast cancer
Shanbeh Zienolddiny, 2013	Norway	nested case–control	rs3749474	563 + 619	PCR	Not mentioned	35 to 74	invasive breast cancer
Anne Grundy,2013	Canada	case–control	rs2035691	437 + 556	SNP Golden Gate (Illumina) assay	Not mentioned	20–80	either in situ or invasive breast cancer
Rabstein, 2014	German	case–control	rs10462028	857 + 852	Sequenom matrix-assisted laser desorption/ionization time-of-flight mass spectrometry	Not mentioned	≤ 80	Breast cancer
Pham, 2019	Korea	case–control	rs374974, rs11133373	941 + 959	High throughput genotyping	Not mentioned	≤ 50y 59.8%; > 50y 40.2%	Breast cancer

**Table 2:** New-Castle Ottawa scale to assess the quality of the included studies

Study	Selection	Selection of controls	Ascertainment of exposure	outcome	Comparability	Outcome Assessment of outcome	Adequate follow-up time	Adequacy of follow up	NOS scores
	Representative of cases				Comparability of the design or analysis				
Aaron E. Hoffman, 2010	1	1	1	1	2	1	0	1	8
Hongji Dai, 2010	0	0	1	1	2	1	0	1	6
Shanbeh Zienolddiny, 2013	0	0	1	1	2	1	0	1	6
Anne Grundy, 2013	0	1	1	1	2	1	0	1	7
Rabstein, 2014	1	1	1	1	2	1	0	1	8
Pham, 2019	1	1	1	1	1	1	0	1	7

### Relationship Between *CLOCK* Polymorphisms and Breast Cancer Risk

**Fig. 2:**
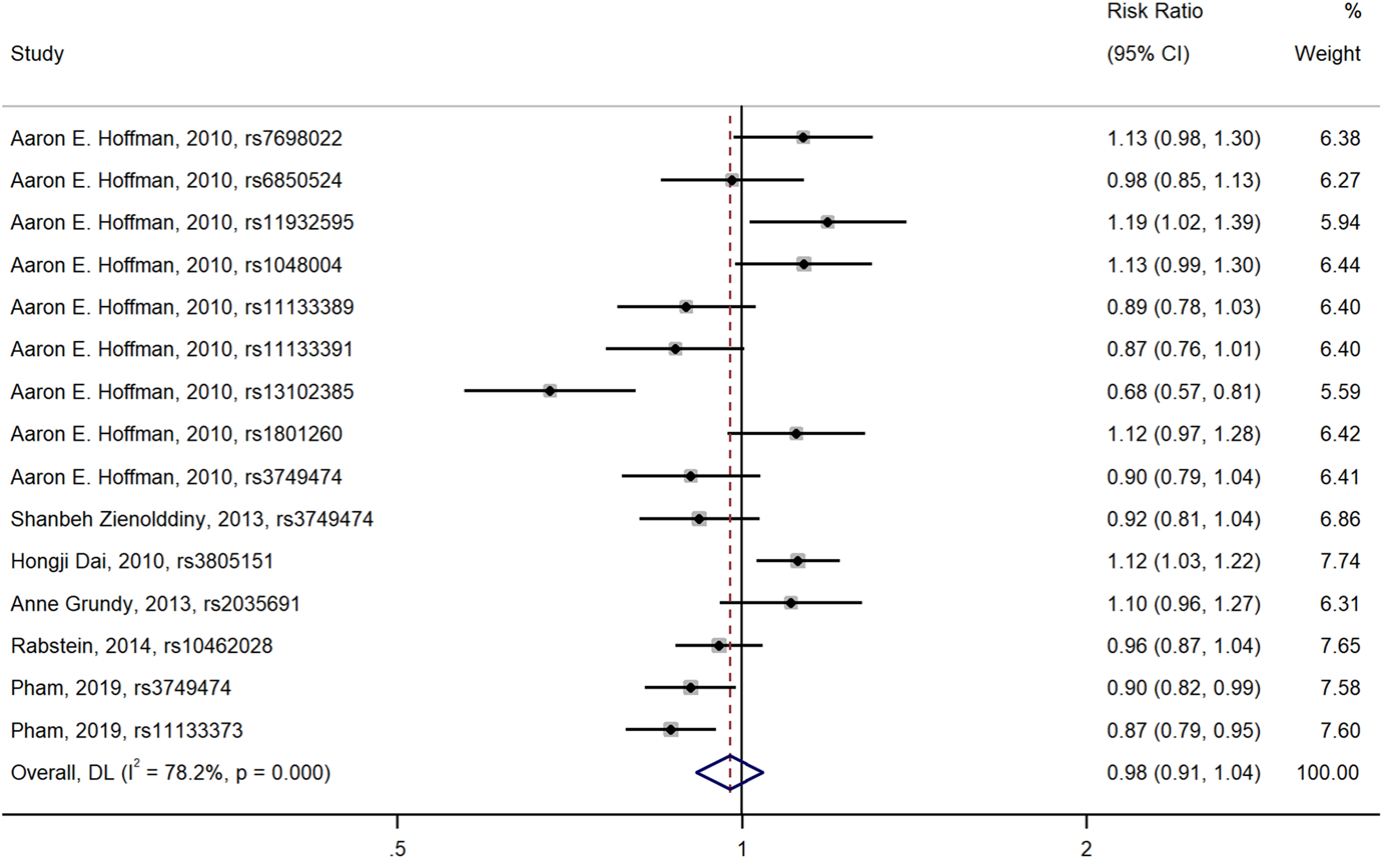
Forest plot of the relationship between polymorphisms in *CLOCK* gene and breast cancer risk (dominant model)

**Fig. 3:**
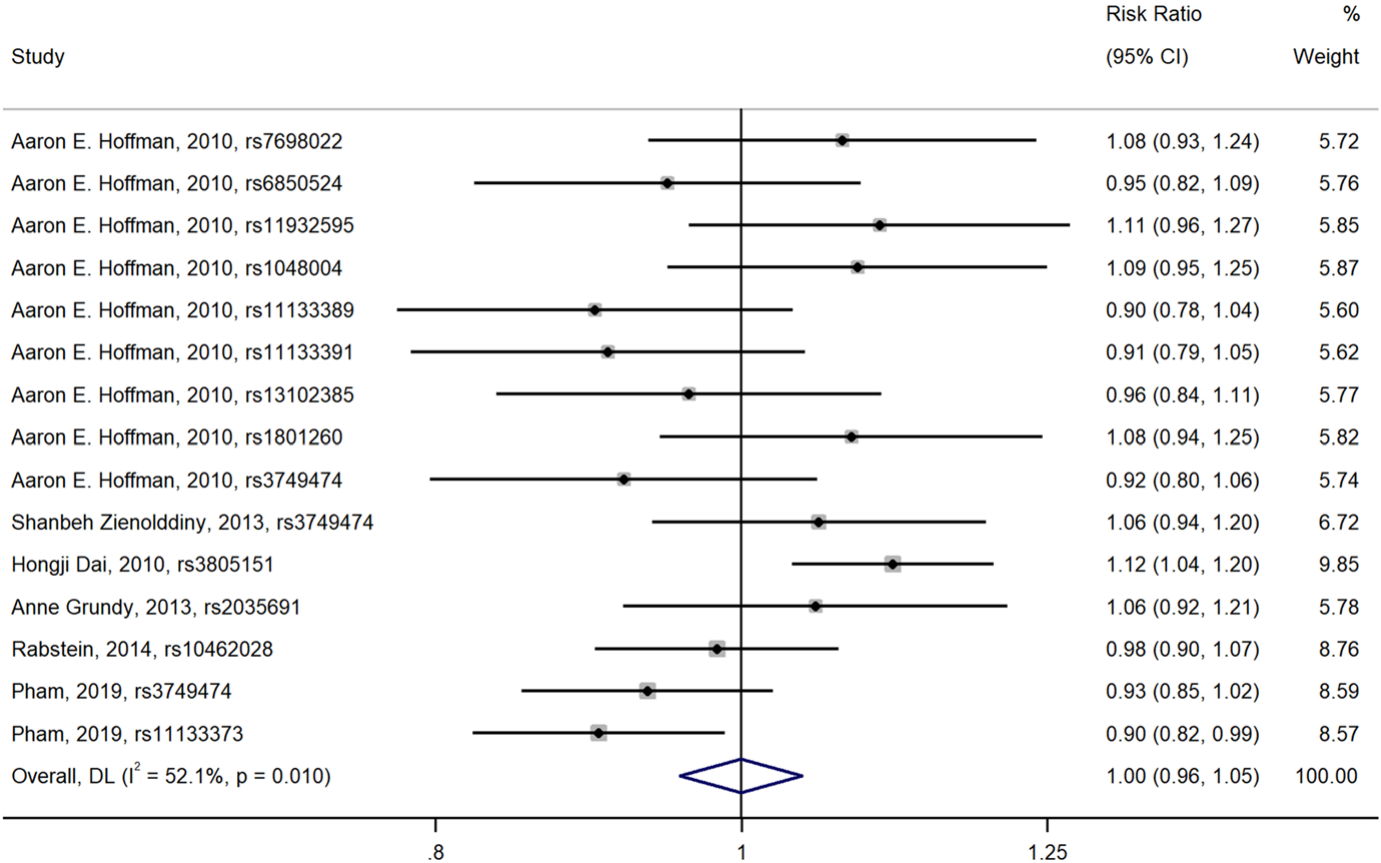
Forest plot of the relationship between polymorphisms in *CLOCK* gene and breast cancer risk (over-dominant model)

**Fig. 4:**
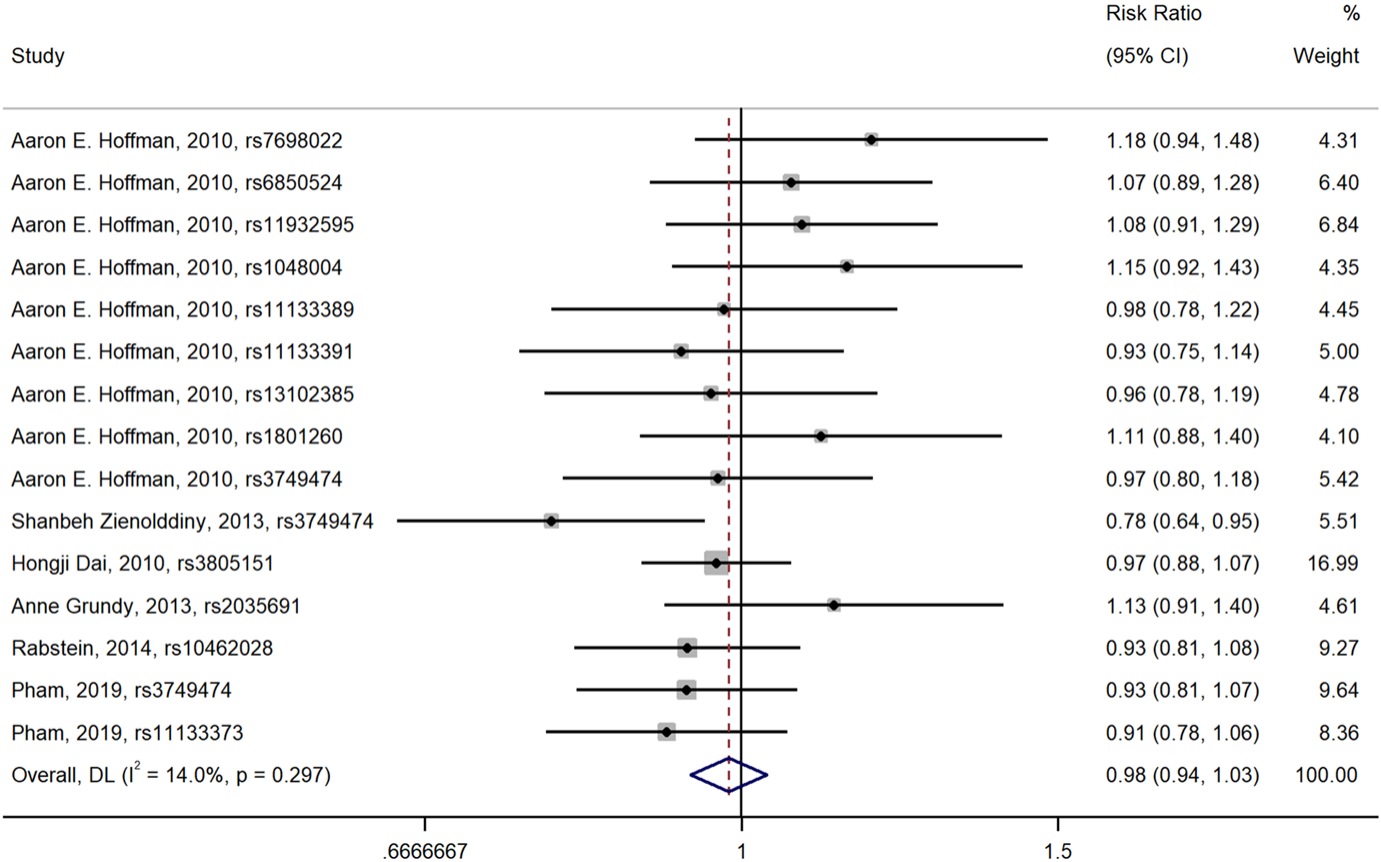
Forest plot of the relationship between polymorphisms in *CLOCK* gene and breast cancer risk (recessive model)

A total of 13 *CLOCK* gene SNPs were included. The dominant model, over-dominant model, and recessive model were used in the meta-analysis. However, we did not find a significant association between *CLOCK* polymorphisms and breast cancer [dominant model: OR (95%CI) = 0.98 (0.91, 1.04), Fig. 2; over-dominant model: OR (95%CI) = 1.00 (0.96, 1.05), Fig. 3; recessive model: OR (95%CI) = 0.98 (0.94, 1.03), Fig. 4].

**Fig. 5:**
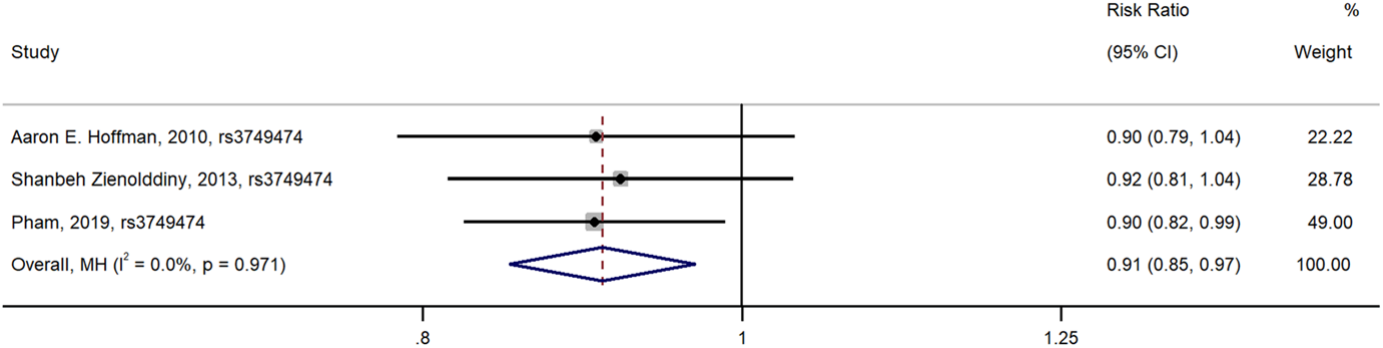
Forest plot of the relationship between polymorphisms in rs3749474and breast cancer risk (dominant model)

A total of three studies examined the association between rs3749474 and breast cancer risk. In the sub-meta-analysis of rs3749474, we found that compared with T/T types of rs3749474, T/C and C/C types of rs3749474 were associated with lower risk of breast cancer [OR (95%CI) = 0.91 (0.85, 0.97), dominant model], with non-significant heterogeneity across studies (I^2^ < 0.001 and _Heterogeneity_ = 0.971). The results of the meta-analysis are shown in Fig. 5.

### Publication Bias

**Fig. 6:**
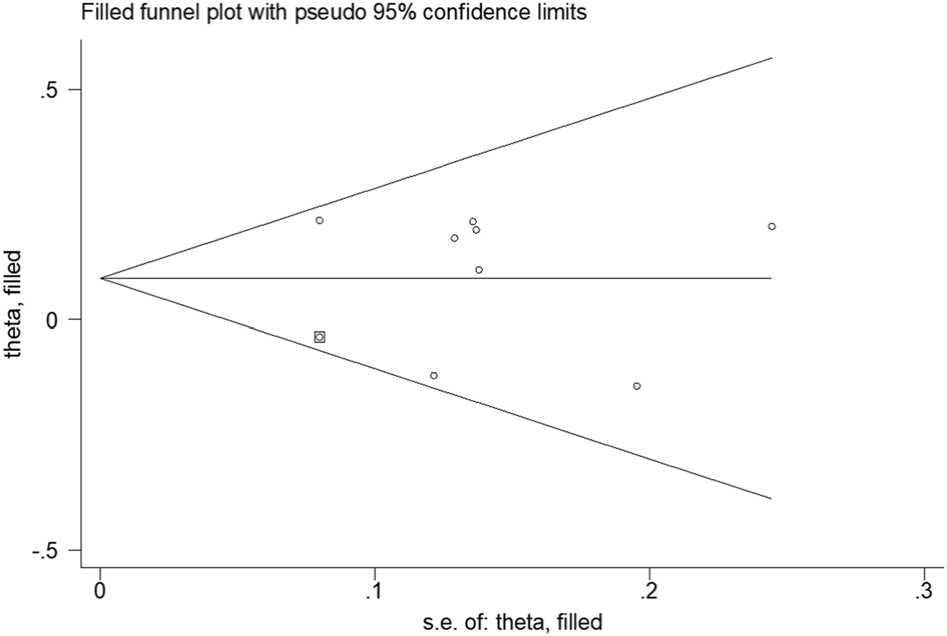
Sensitivity analysis of the relationship between polymorphisms in *CLOCK* gene and breast cancer risk

**Fig. 7:**
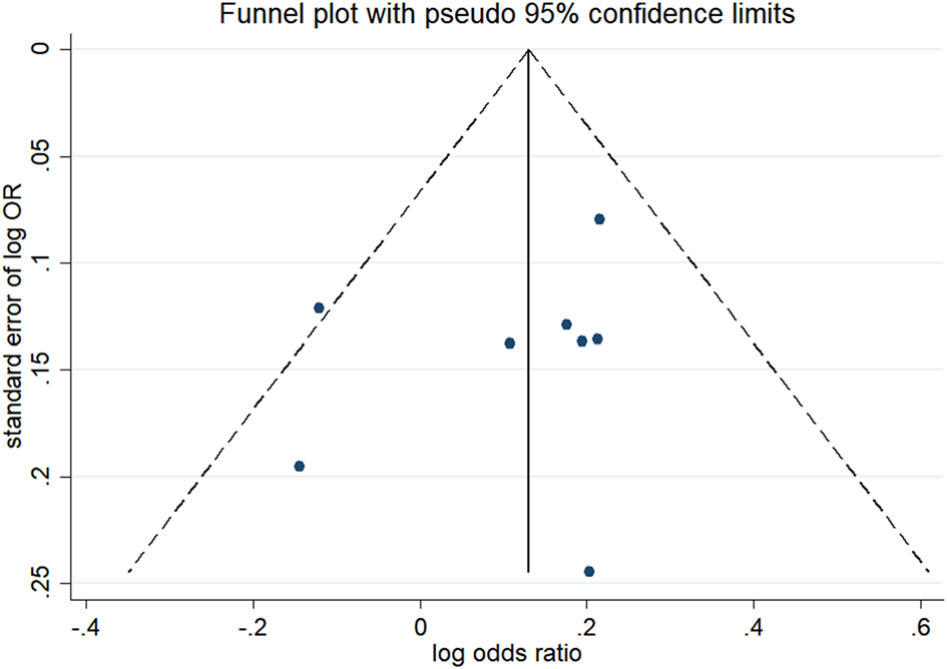
Cutting and patching method of the relationship between polymorphisms in *CLOCK* gene and breast cancer risk

The Cochrane Collaboration’s tool for assessing the risk of bias is shown in Fig. 6. As expected, only the two full-paper trials were of high quality. The funnel diagram is shown in Fig. 7. Harbord's regression test (*P* = 0.864) indicated no publication bias.

### Sensitivity Analysis

**Fig. 8:**
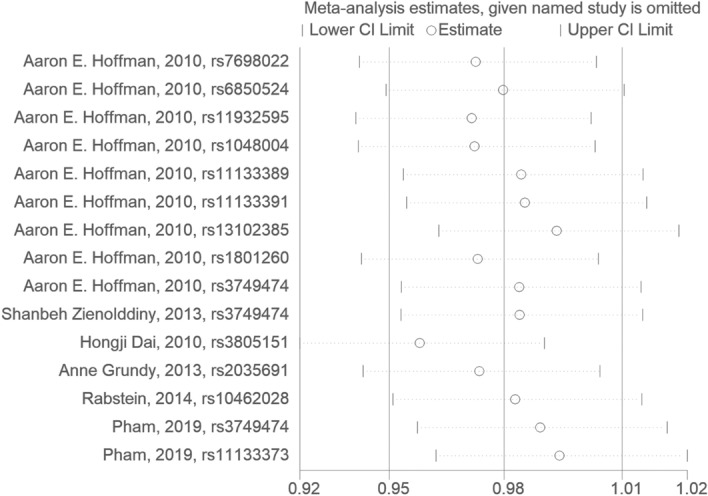
Funnel Plot for publication bias

The leave-one-out sensitivity analysis showed that no single study significantly affected the pooled correlation from the meta-analysis (the OR ranging from 0.98 to 1.24), which indicated the reliability of the findings. The trim and fill method suggests funnel plot symmetry (Fig. 8).

## Discussion

To provide insight into the relationship between *CLOCK* polymorphisms and breast cancer risk, we performed a meta-analysis among 10,164 participants, including 5488 breast cancer cases and 4676 controls. We found that the mutation types of one *CLOCK* SNP, rs3749474 were significantly associated with the risk of breast cancer. Compared with T/T types of rs3749474, T/C and C/C types of rs3749474 were associated with a lower risk of breast cancer [OR (95%CI) = 0.93 (0.88, 0.98)]. These results uncover the important role of *CLOCK* polymorphisms in breast cancer.

Breast cancer is one of the most common cancer types among women. Further understanding of progress, treatments, and biomarkers of breast cancer is urgently needed. A number of risk factors have been found to be associated with the risk of breast cancer these years, including cytokine, miRNA, and trace elements in food, etc. (Çetin and Topçul 2022; Cheng et al. 2022; Majed 2022; Wang et al. 2022; Xin and Zhiyuan 2022; Zhou et al. 2022). Based on these, molecular markers and reference laboratory tests for breast cancer diagnosis and prognosis have been developed, but the methods are limited to specific subtypes (Park et al. 2017). Thus, we still need novel approaches to the diagnosis of breast cancer.

It has been reported that disruption in sleep and circadian rhythm disorders are significantly associated with the risk of breast cancer (He et al. 2015). An animal study by Zhang et al. showed that disturbances of the circadian system through ablation of the pineal gland or constant light exposure could result in breast carcinogenesis (Zhang et al. 2020). In humans, a meta-analysis of 26 studies with more than one million participants found that female flight attendants who worked long shifts at night had an increased incidence of breast cancer (Manouchehri et al. 2021). The mechanisms may lie in the secretion disorder of nocturnal melatonin in the pineal gland, the activities of the hormone estrogen and estrogen receptor, and the circadian clock genes (Stevens 2005; Stevens and Davis 1996; Stevens and Rea 2001).

Evidence has also shown that individual genes of the circadian clock play a role in controlling tumorigenesis. *CLOCK* is one of the core circadian clock genes. Its corresponding protein belongs to the basic helix-loop-helix PAS family of transcription factors and forms heterodimers with BMAL1 to enhance target gene expression (Benna et al. 2017). *CLOCK* has been identified as a significant modifier of breast cancer incidence (Sancar and Gelder 2021). On the one hand, the expression of oncogene *c-Myc* could be controlled by *CLOCK*; on the other hand, oncogenes *c-Myc*, *P53*, and *Ras* could affect the face of *CLOCK* (Sancar and Gelder 2021). Moreover, *CLOCK* could induce remodeling of the tumor microenvironment cells by disturbing the cellular metabolism, altering gene expression, and aberrantly activating signaling pathways (Malla et al. 2021). Using state-of-the-art immune cell deconvolution and pathway quantification, Wu et al. demonstrated that abnormal expression of *CLOCK* contributed to T cell exhaustion and global upregulation of immune inhibitory molecules (Wu et al. 2019).

SNP rs3749474 is located in the *CLOCK* 3’-untranslated region. In the current study, we performed the sub-group meta-analysis only for rs3749474, and we found that compared with wild type T/T, the homozygous mutant-type C/C and heterozygous-type T/C of rs3749474 were associated with a lower risk of breast cancer. However, rs3749474 is not specific to breast cancer. Zhou et al. found that the homozygous mutant-type of rs3749474 was associated with better survival of colorectal cancer (F. Zhou et al. 2012). It has also been reported that the rs3749474 polymorphism could modulate the effect of energy intake on nutritional status (Camblor Murube et al. 2020; Espinosa-Salinas et al. 2020). The biological role of rs3749474 on breast cancer still needs further investigation.

To our knowledge, this is the first meta-analysis of the association between *CLOCK* polymorphisms and breast cancer risk, which incorporates multiple SNPs. Our findings may gain insights into breast cancer genetics and uncover the roles of the circadian clock gene on breast cancer, which helps provide a basis for further research in breast cancer diagnosis and treatment. However, the present study has some limitations. First, we could not conduct a subgroup meta-analysis given the limited data in the included studies, so our results should be interpreted cautiously. Second, only studies published in English were included; therefore, we might have missed some relevant studies in other languages. Third, bias might have been introduced due to confounding factors among different studies.

## Conclusion

The mutation types of *CLOCK* gene rs3749474 are negatively associated with the risk of breast cancer. More studies are still needed to confirm this conclusion.

## Data Availability

The datasets generated during and/or analyzed during the current study are available from the corresponding author on reasonable request.
